# Supplementary material for: Regulatory T cells expressing CD19-targeted chimeric antigen receptor restore homeostasis in Systemic Lupus Erythematosus
Source: Nat Commun. 2024 Mar 27;15:2542. doi: 10.1038/s41467-024-46448-9 (PMC10973480; doi:10.1038/s41467-024-46448-9)
Supplement: Supplementary file 1 — Supplementary Information [file 41467_2024_46448_MOESM1_ESM.pdf]

Manuscript title:

"Regulatory T cells expressing CD19-targeted chimeric antigen receptor restore homeostasis in Systemic Lupus Erythematosus"

Doglio M.[1*], Ugolini A.[1], Bercher-Brayer C.[1], Camisa B.[1], Toma C.[1], Norata R.[2], Del Rosso S.[3], Greco R.[4], Ciceri F.[4], Sanvito F.[2,5], Casucci M.[6], Manfredi A.A.[7], Bonini C.[1*]

Affiliations:
1 Experimental Hematology Unit, Division of Immunology Transplantation and Infectious Diseases (DITID), IRCCS San Raffaele Scientific Institute, Milan, Italy
2 GLP Test Facility, San Raffaele Telethon Institute for Gene Therapy (SR-Tiget), IRCCS San Raffaele Scientific Institute, Milan, Italy
3 Autoimmunity Lab, IRCCS San Raffaele Hospital, Milan, Italy
4 Hematology and Bone Marrow Transplantation Unit, IRCCS San Raffaele Hospital, Milan, Italy
5 Pathology Unit, Division of Experimental Oncology, IRCCS San Raffaele Scientific Institute, Milan, Italy
6 Innovative Immunotherapies Unit, Division of Immunology Transplantation and Infectious Diseases (DITID), IRCCS San Raffaele Scientific Institute, Milan, Italy
7 Autoimmunity and Vascular Inflammation Unit, Division of Immunology Transplantation and Infectious Diseases (DITID), IRCCS San Raffaele Scientific Institute, Milan, Italy
* Corresponding authors. E-mail: bonini.chiara@hsr.it, doglio.matteo@hsr.it

Gating strategy

Example of Treg phenotype

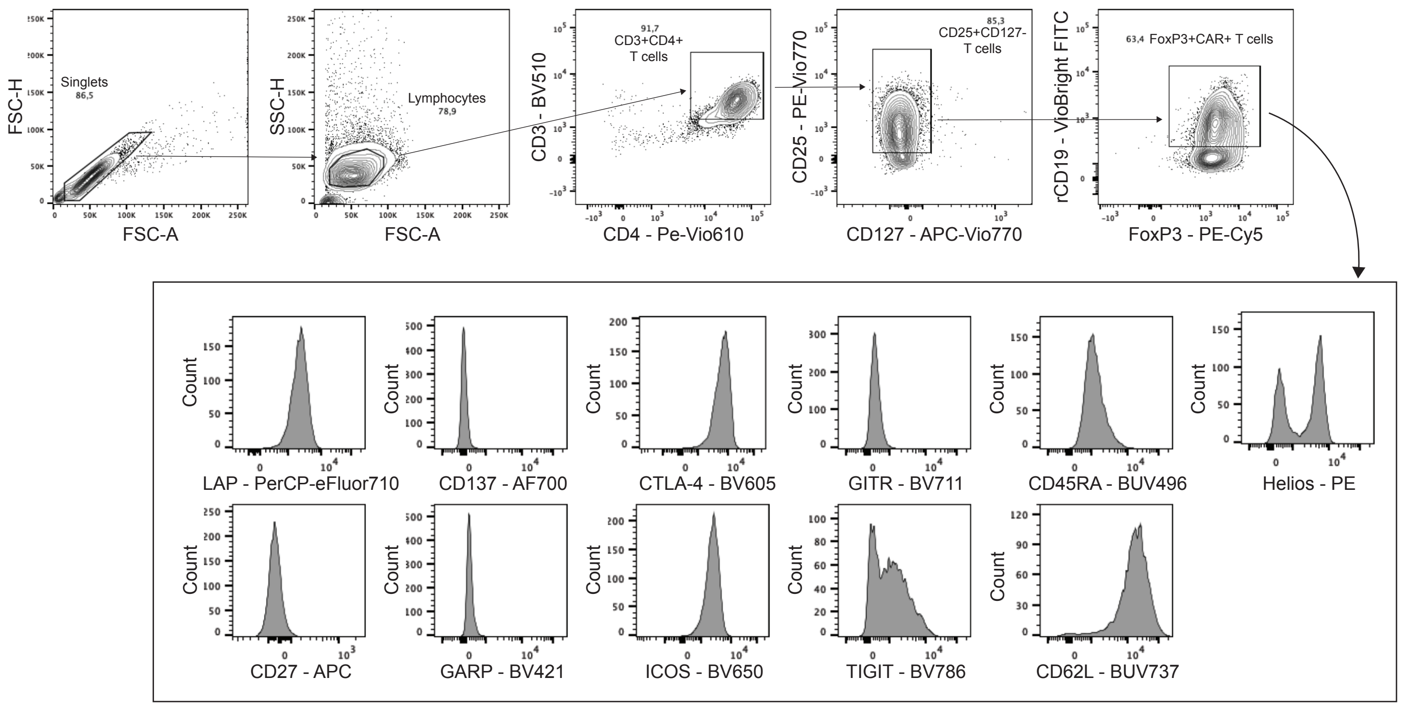

Example of mouse bleeding

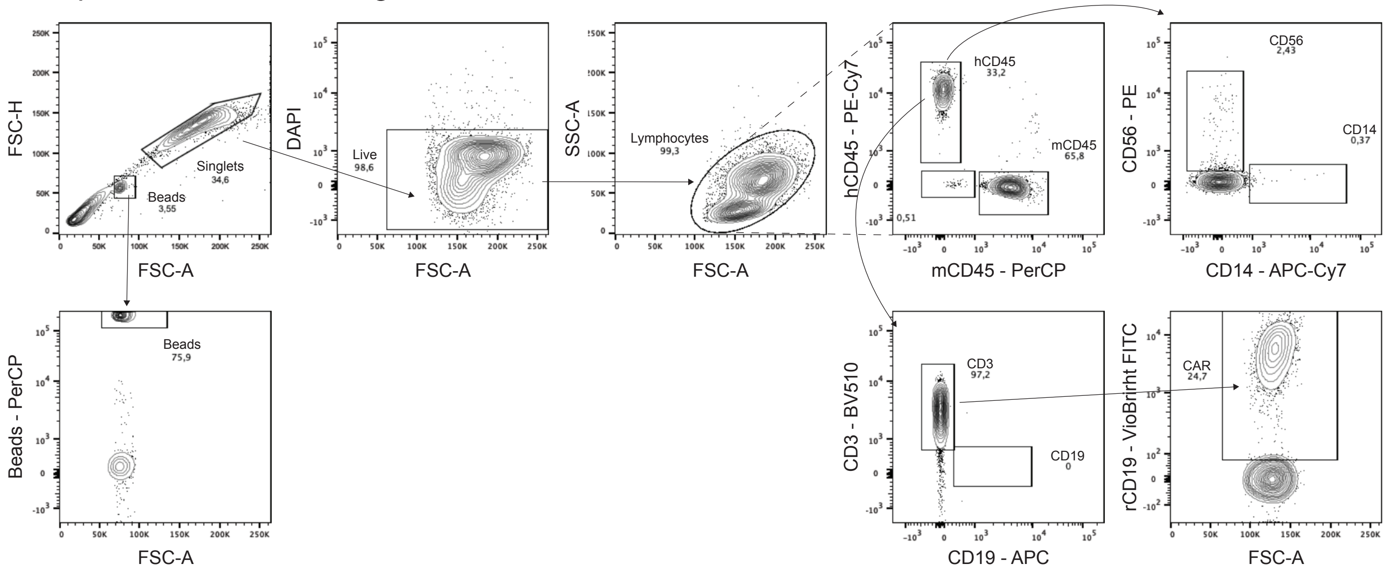

Example of mouse organ analysis

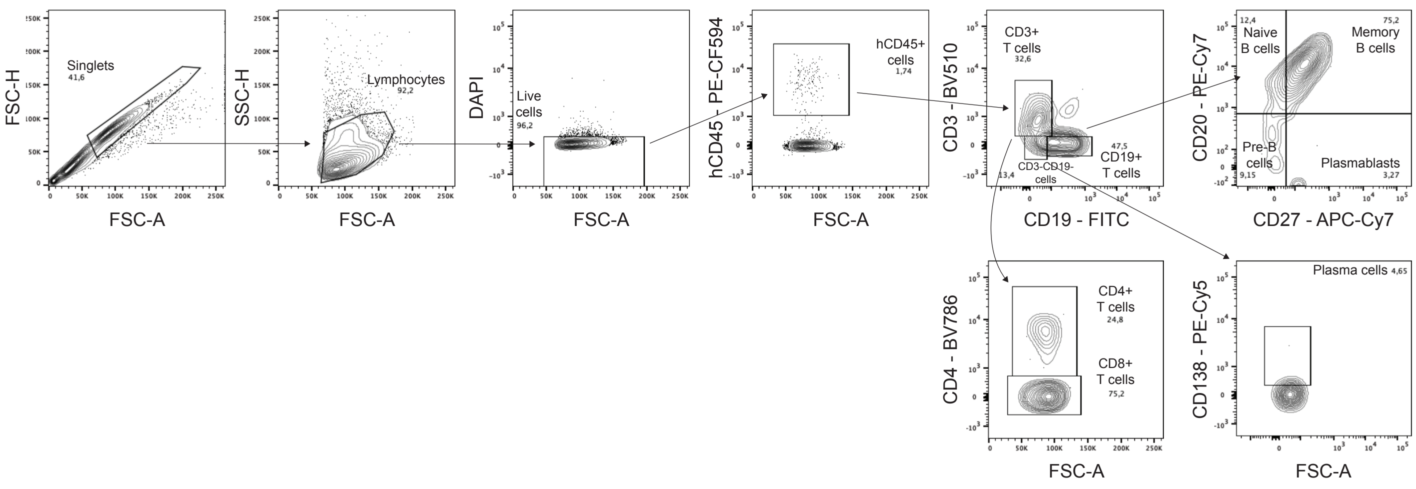

Supplementary Figure 1

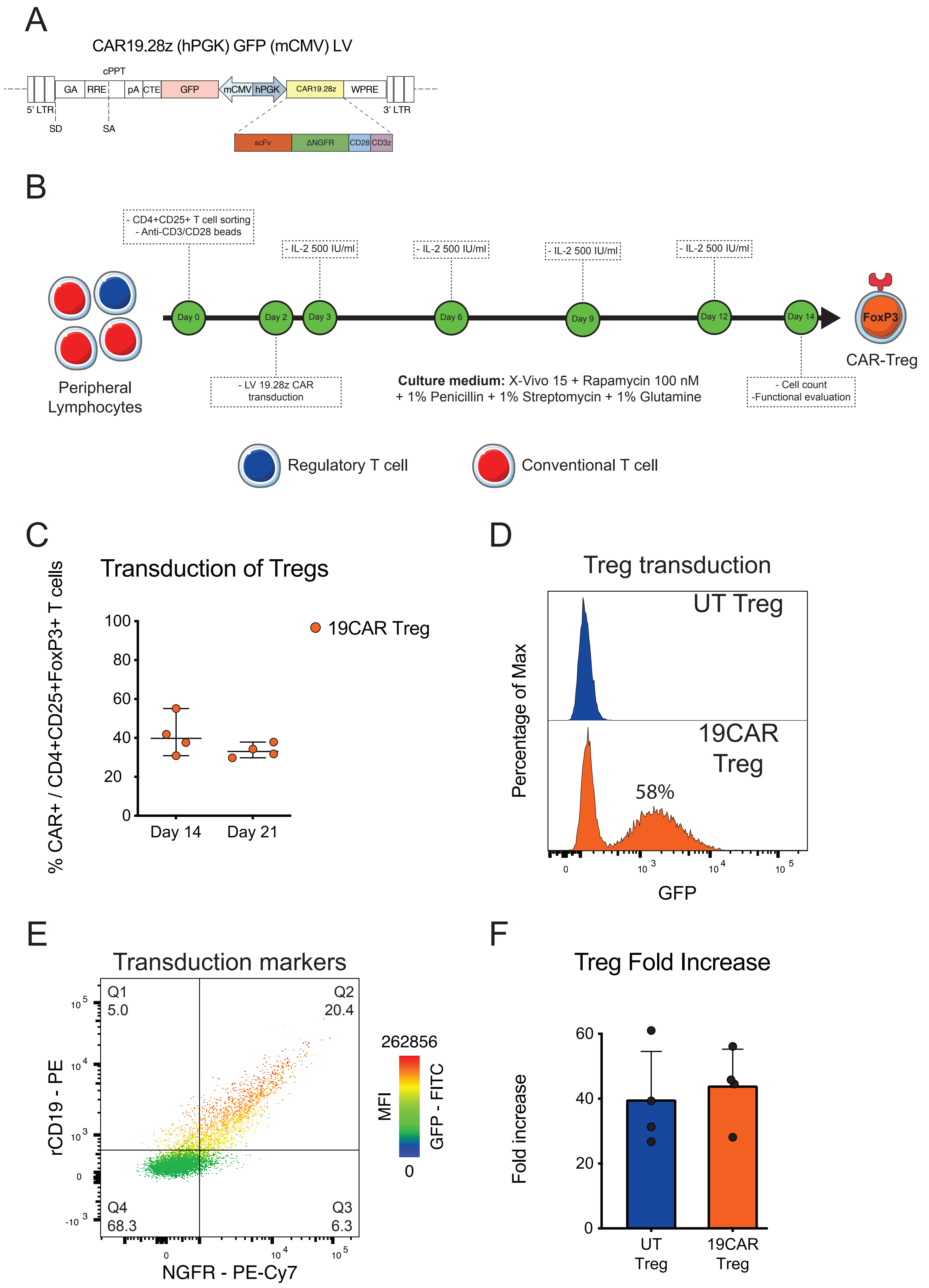

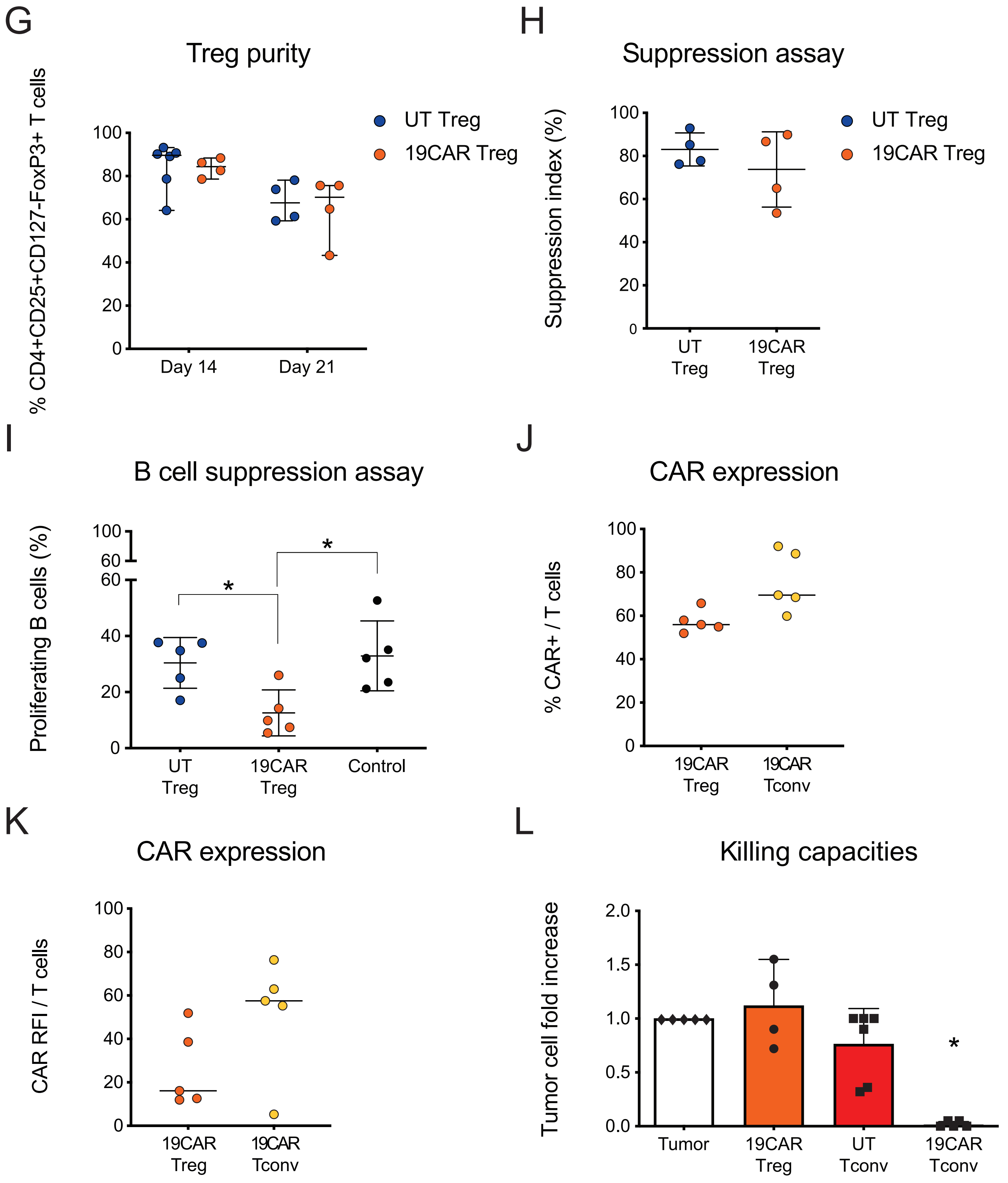

Supplementary Figure 1 – 19CAR-Treg feasibility and efficacy

A. CAR19.28z bi-directional lentiviral vector (LV) schematic representation. In sense, the anti-CD19 second-generation CAR is under a Phosphoglycerate Kinase (PGK) promoter. In antisense, GFP is under a minimal CMV promoter (mCMV). Other LV components: LTR long terminal repeat, SD splice donor, SA splice acceptor, GA gag-pol element, RRE REV responsive element, cPPT central polypurine tract, pA polyadenilation signal, CTE constitutive transport element, WPRE woodchuck hepatitis virus post-transcriptional regulatory element.

B. Schematic representation of the CAR-Treg generation protocol.

C. Treg transduction with CAR19.28z LV, assessed by evaluating the NGFR expression on CD3+CD4+CD25+CD127-FoxP3+ cells. N=4. Paired Wilcoxon test for non-parametric variables.

D. Representative plot of 19CAR-Treg transduction, assessed evaluating the GFP expression.

E. CAR expression assessed with the recombinant CD19 (rCD19) in PE, anti-NGFR (Nerve Growth Factor Receptor) antibody in PE-Cy7 and GFP (Green Fluorescent Protein). The Mean Fluorescence Intensity of the GFP signal is reported as a color gradient, ranging from 0 (blue) to 262856 (red).

F. Expansion rate of UT and 19CAR-Tregs at day +14. Results are expressed as fold increase. N=4. Mann-Whitney test.

G. Percentage of regulatory T cells among T lymphocytes in either UT- or 19CAR-Tregs at day +14 and at day +21, defined as CD3+CD4+CD25+CD127-FoxP3+ cells. N=6. Mann-Whitney test.

Supplementary Figure 1 – 19CAR-Treg feasibility and efficacy (continued)

H. Engineered Treg suppressive capacities. Results are expressed as Suppression Index, calculated as Suppression index = [1-(PBMCs' proliferation with Tregs)/(PBMCs' proliferation alone)] * 100. N=4. Student's T-test.

I. CAR-Treg antigen-specific suppressive capacities. Results are expressed as percentages of proliferating B lymphocytes. N = 5 for each group. One-way ANOVA with Tukey correction for multiple comparison. * = p-value <0.05.

J-K. CAR expression in both 19CAR-Tregs and 19CAR-Tconvs employed for the functional assay reported in Suppl. Fig. 1L reported as percentage of CAR+ cells and CAR relative fluorescence intensity (RFI) relative to the untransduced cells. N = 5 for each group.

L. Killing capacities of different lymphocyte subpopulations. Results are expressed as tumor fold increase. N=6 for CD19+ tumor cells, UT- and 19CAR-Tconvs. N=4 for 19CAR-Tregs. One-way ANOVA with Tukey correction for multiple comparison. * = p-value <0.05.

All the results are expressed as mean ± standard deviation.

Supplementary Figure 2

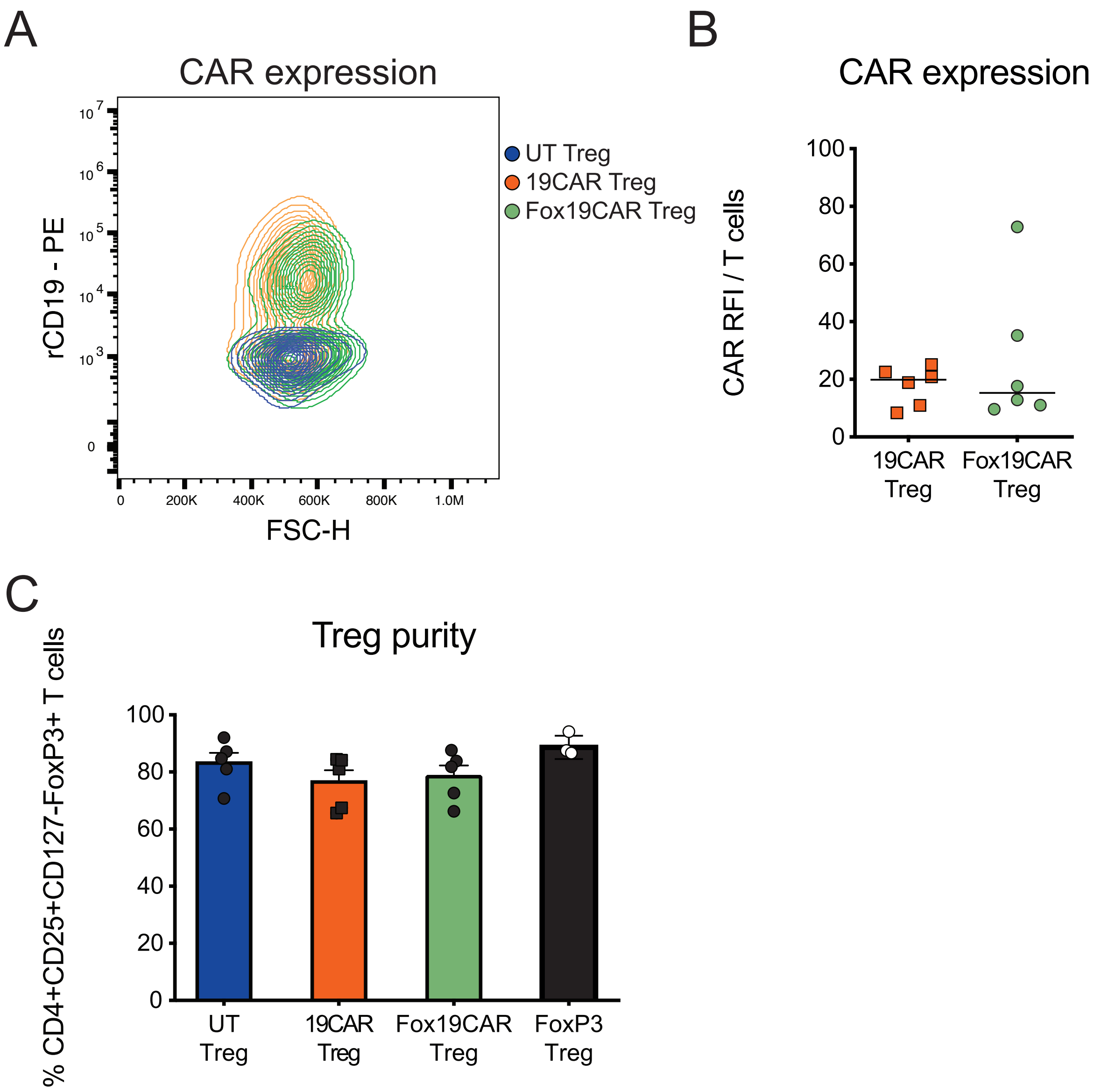

Supplementary Figure 2 – Fox19CAR-Treg validation

A. Representative plot of CAR transduction. The contour plot showed the CAR expression in UT- (blue), 19CAR- (orange) and Fox19CAR-Tregs (green), detected with recombinant CD19 (rCD19) in PE.

B. CAR expression in both 19CAR- and Fox19CAR-Tregs. The CAR expression on engineered cells employed for the functional assay reported in Fig. 1F was assessed with the rCD19 by flow cytometry and expressed as CAR relative fluorescence intensity (RFI) relative to the untransduced cells. N = 6 for each group.

C. Percentage of regulatory T cells among T lymphocytes in UT-, 19CAR-, Fox19CAR- or FoxP3-Tregs at day +21. Regulatory T cells were defined as CD3+CD4+CD25+CD127-FoxP3+ cells and were identified by flow cytometry. The results are expressed as mean ± standard deviation. N=5 for UT-, Fox19CAR- and 19CAR-Tregs. N=3 for FoxP3-Tregs. One-way ANOVA with Tukey correction for multiple comparison.

Supplementary Figure 3

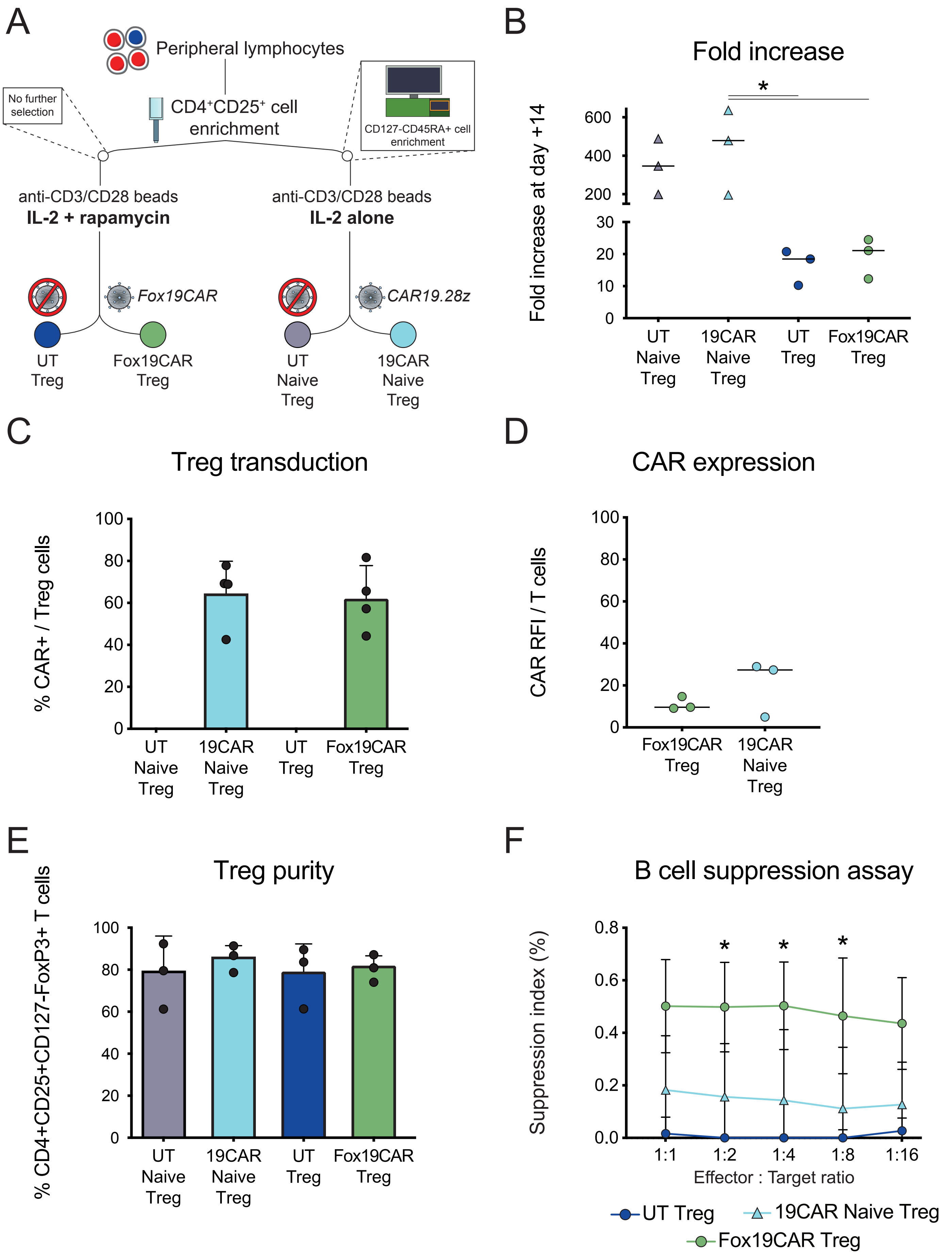

G

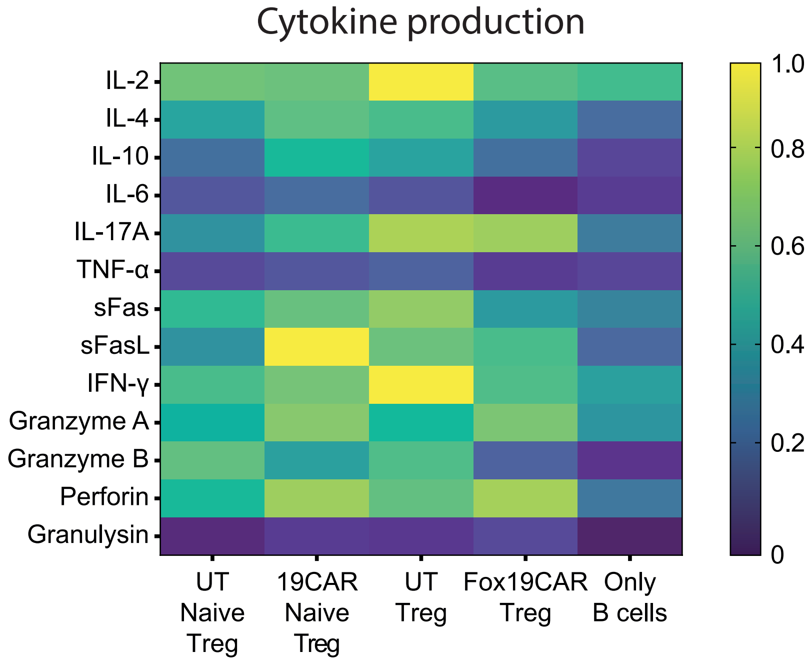

Supplementary Figure 3 – Foxx19CAR-Tregs outperform naïve-derived 19CAR-Tregs in suppressing proliferating autologous B lymphocytes

A. Schematic representation of naïve- or CD4+CD25+-derived Treg engineering with two different LV vectors.

B. Expansion rate of naïve- or CD4+CD25+-derived Tregs transduced with CAR19.28z LV or Fox19CAR LV, respectively, or left untransduced, assessed at day +14, expressed as fold increase. N=3 for each group. One-way ANOVA with Tukey correction for multiple comparison. * = p-value <0.05.

C. Transduction efficiency of CAR19.28z LV and Fox19CAR LV on naïve- or CD4+CD25+-derived Tregs measured as percentage of CAR+ cells among CD4+CD25+CD127-FoxP3+ lymphocytes at day +14. Recombinant CD19 (rCD19) was employed as transduction markers to estimate the percentage of CAR+ cells. N=4 for each group. One-way ANOVA with Tukey correction for multiple comparison.

D. CAR expression in both naïve- and CD4+CD25+-derived CAR-Tregs employed for the functional assay reported in Suppl. Fig. 1F assessed with the rCD19 and expressed as CAR relative fluorescence intensity (RFI) relative to the untransduced cells. N = 3 for each group.

E. Percentage of regulatory T cells among T lymphocytes in either naïve- or CD4+CD25+-derived Tregs transduced with CAR19.28z LV or Fox19CAR LV, respectively, or left untransduced, assessed at day +21. Tregs were defined as CD3+CD4+CD25+CD127-FoxP3+ cells. N=3. One-way ANOVA test with Tukey correction for multiple comparisons.

F. Antigen-specific suppressive capacities of naïve- or CD4+CD25+-derived Tregs transduced with CAR19.28z LV or Fox19CAR LV, respectively, or left untransduced. Results are expressed as Suppression index = [1-(B cell proliferation with Tregs)/(B cell proliferation alone)] * 100. N=3 for each group. Two-way ANOVA with Tukey correction for multiple comparison. * = p-value <0.05.

G. Heat map reporting the cytokine profile secreted by engineered or untransduced Tregs upon antigen-specific stimulation. The amount of each cytokine was normalized across the various groups, scaled to range from 0 (minimum) to 1 (maximum), and a color gradient was generated. For each group, the relative abundance of each cytokine is reported. N=3 for each group. Kruskall-Wallis test.

All the results are expressed as mean ± standard deviation.

Supplementary Figure 4

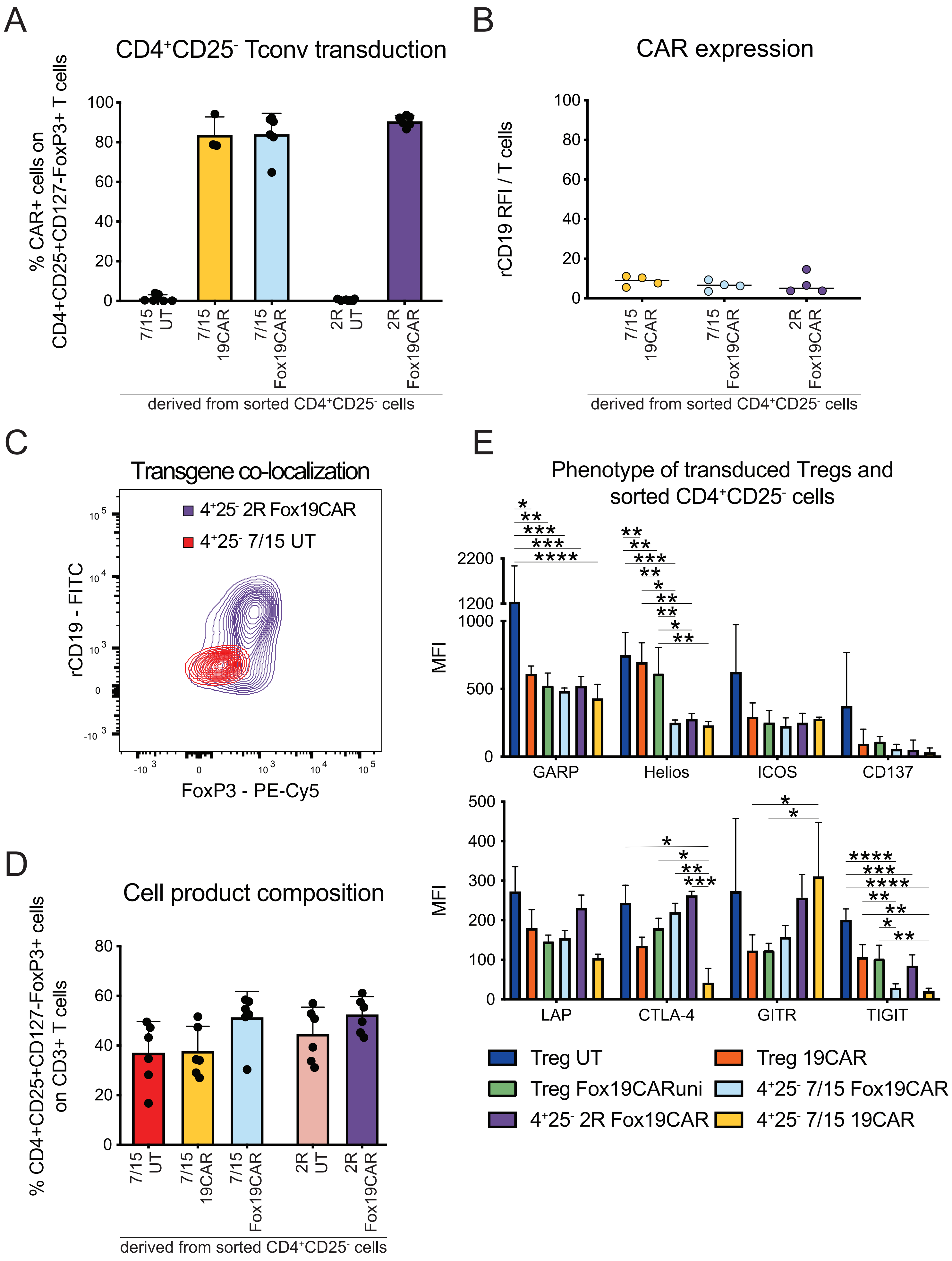

F

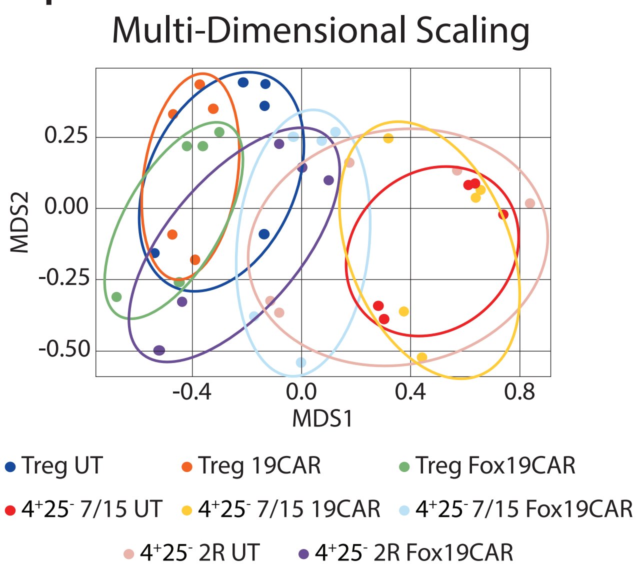

Supplementary Figure 4 – CD4+CD25- engineered cell phenotypic characterization

A. Transduction efficiency of CD4+CD25- engineered cells with either CAR19.28z LV or Fox19CAR at day +14. CAR+ cells were assessed by using recombinant CD19 (rCD19). N=6 for CD4+CD25- 7/15 UT, 7/15 Fox19CAR, 2R UT and 2R Fox19CAR. N=3 for CD4+CD25- 7/15 19CAR. One-Way ANOVA test with Tukey correction for multiple comparison.

B. CAR expression in CD4+CD25- engineered cells with either CAR19.28z LV or Fox19CAR LV. The CAR expression on transduced lymphocytes employed for the functional assay reported in Fig. 2D was assessed with the rCD19 by flow cytometry and expressed as CAR relative fluorescence intensity (RFI) relative to the untransduced cells. N = 4 for each group.

C. Representative flow cytometry plot for co-localization of CAR and FoxP3 in 2R Fox19CAR and 7/15 UT cells obtained from CD4+CD25- sorted lymphocytes. CAR expression was assessed by flow cytometry at day +14 using the recombinant CD19 (rCD19).

D. Percentage of CD4+CD25+CD127-FoxP3+ cells measured at day +21 in the different cellular products obtained upon manipulation of CD4+CD25- cells, culture in IL-2 and rapamycin (2R)- or IL-7 and IL-15 (7/15)- supplemented media, and transduced with CAR19.28z, Fox19CAR LV or left untreated. N=6 for each group. One-way ANOVA test with Tukey correction for multiple comparison.

E. Evaluation of the principal Treg-associated markers on the cellular products originated from sorted CD4+CD25+ (Tregs) and CD4+CD25- cells. Marker expressions were assessed by flow cytometry after 21 days of culture and reported as Mean Fluorescent Index (MFI). N = 3 for each group. One-way ANOVA test with Tukey correction for multiple comparison. * = p-value <0.05, ** = p-value <0.01, *** = p-value <0.001, **** = p-value <0.0001.

F. Principal Component Analysis (PCA) showing the phenotype similarity of different samples derived from either engineered or untransduced Tregs and CD4+CD25--derived cells. Samples were analyzed by multi-parametric flow cytometry. Similarity was calculated employing cytoChain36 according to the similarity of expression of the evaluated markers. MDS = multi-dimensionality scaling. N=5 for each group.

All the results are expressed as mean ± standard deviation.

Supplementary Figure 5

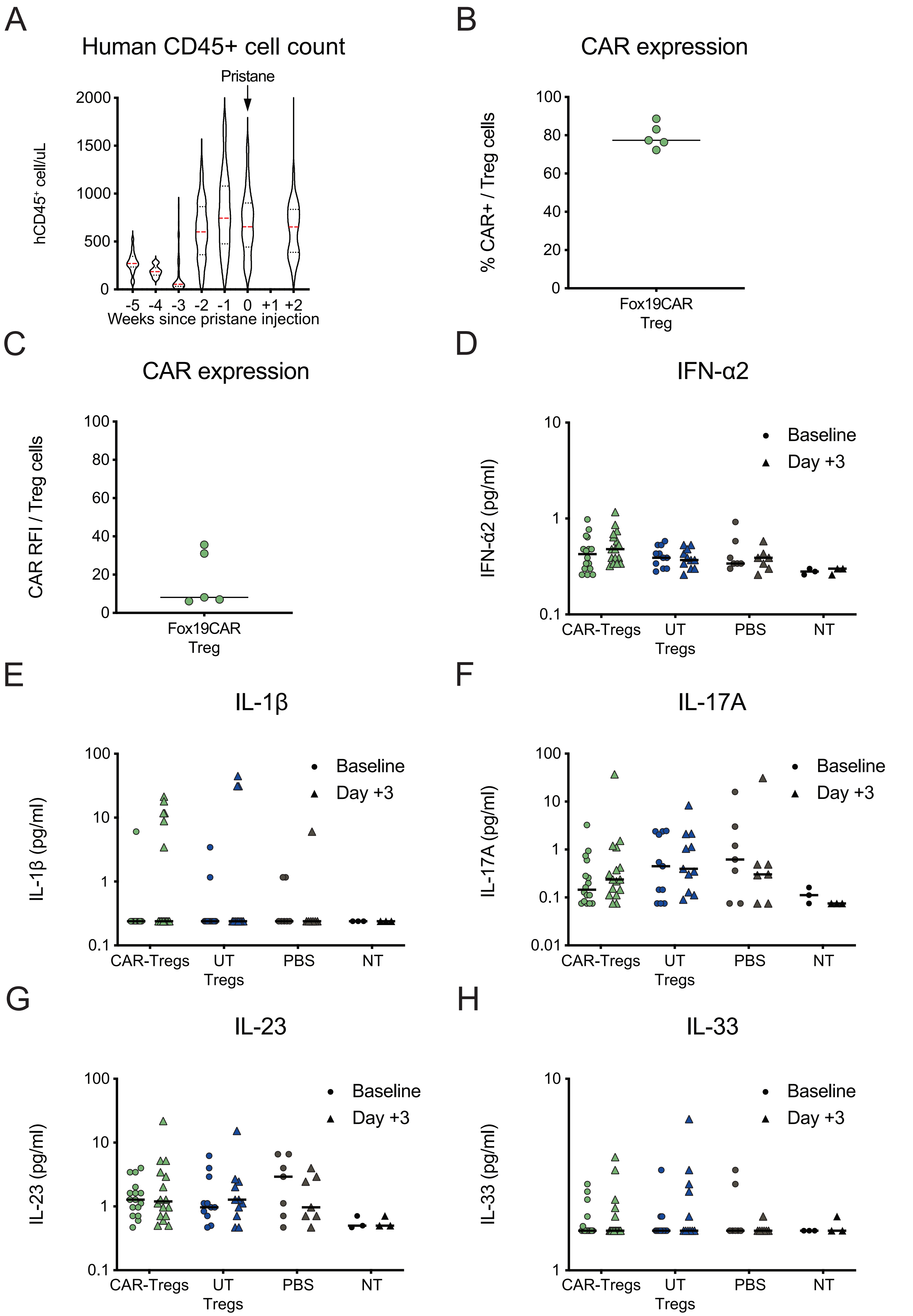

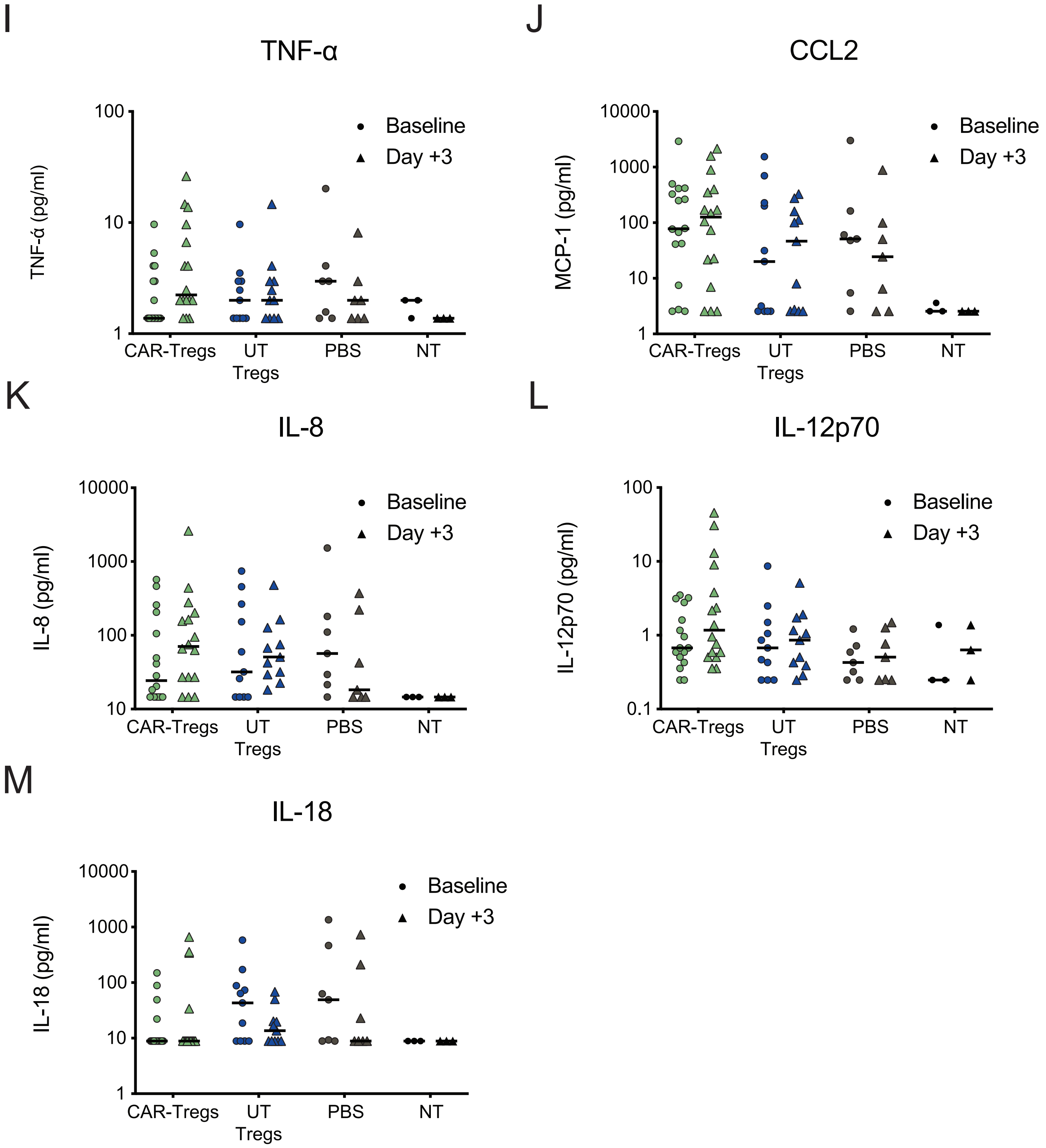

Supplementary Figure 5 – Circulating pro-inflammatory cytokines in a humanized mouse model of SLE

A. Longitudinal assessment of circulating human leukocytes, identified as hCD45+ cells. Pristane injection is indicated with an arrow. N=53. One-way ANOVA with Tukey correction for multiple comparison.

B-C. Fox19CAR-Treg CAR expression. The CAR expression on engineered Tregs employed for the in vivo experiment reported in Fig. 3-4 was assessed with the rCD19 by flow cytometry and expressed as percentage of CAR+ cells and CAR relative fluorescence intensity (RFI) relative to the untransduced cells. N = 5 for each group.

Mean IFN-a2 **(D)**, IL-1b **(E)**, IL-17A **(F)**, IL-23 **(G)**, IL-33 **(H)**, TNF-a **(I)**, CCL2 **(J)**, IL-8 **(K)**, IL-12p70 **(L)**, and IL-18 **(M)** levels in peripheral blood before and 3 days after the injection of Fox19CAR-Treg, UT Tregs or PBS. As control, humanized mice not treated with pristane (NT) are included. Cytokine levels were assessed employing a bead-based immunoassay (Biolegend Legendplex 13-plex kit) according to the manufacturer's instructions. Each dot represents a single mouse. N=37 (16 CAR-Tregs, 11 UT-Tregs, 7 PBS, 3 NT). Paired Wilcoxon test for non-parametric data.

Supplementary Figure 6

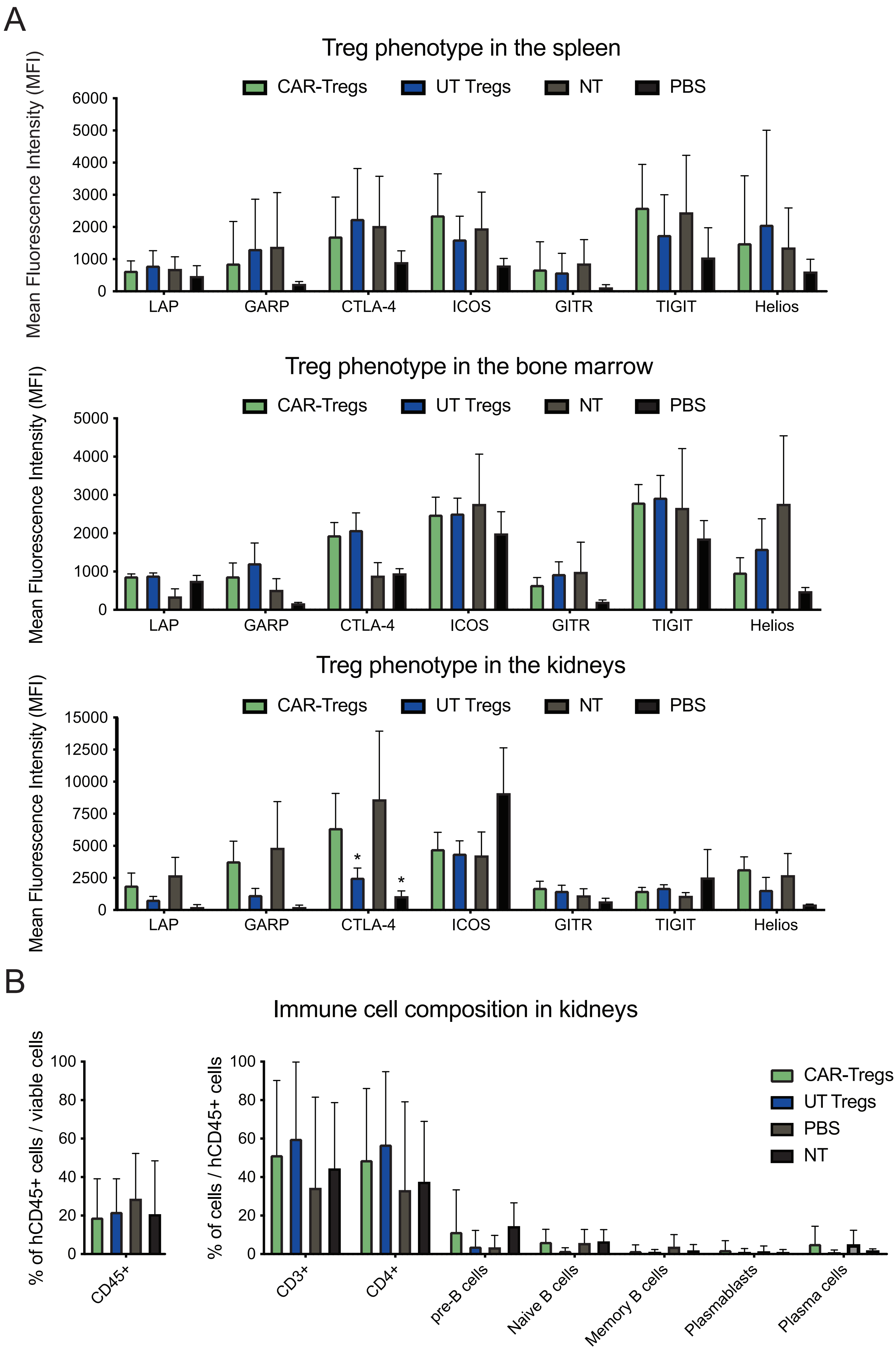

C

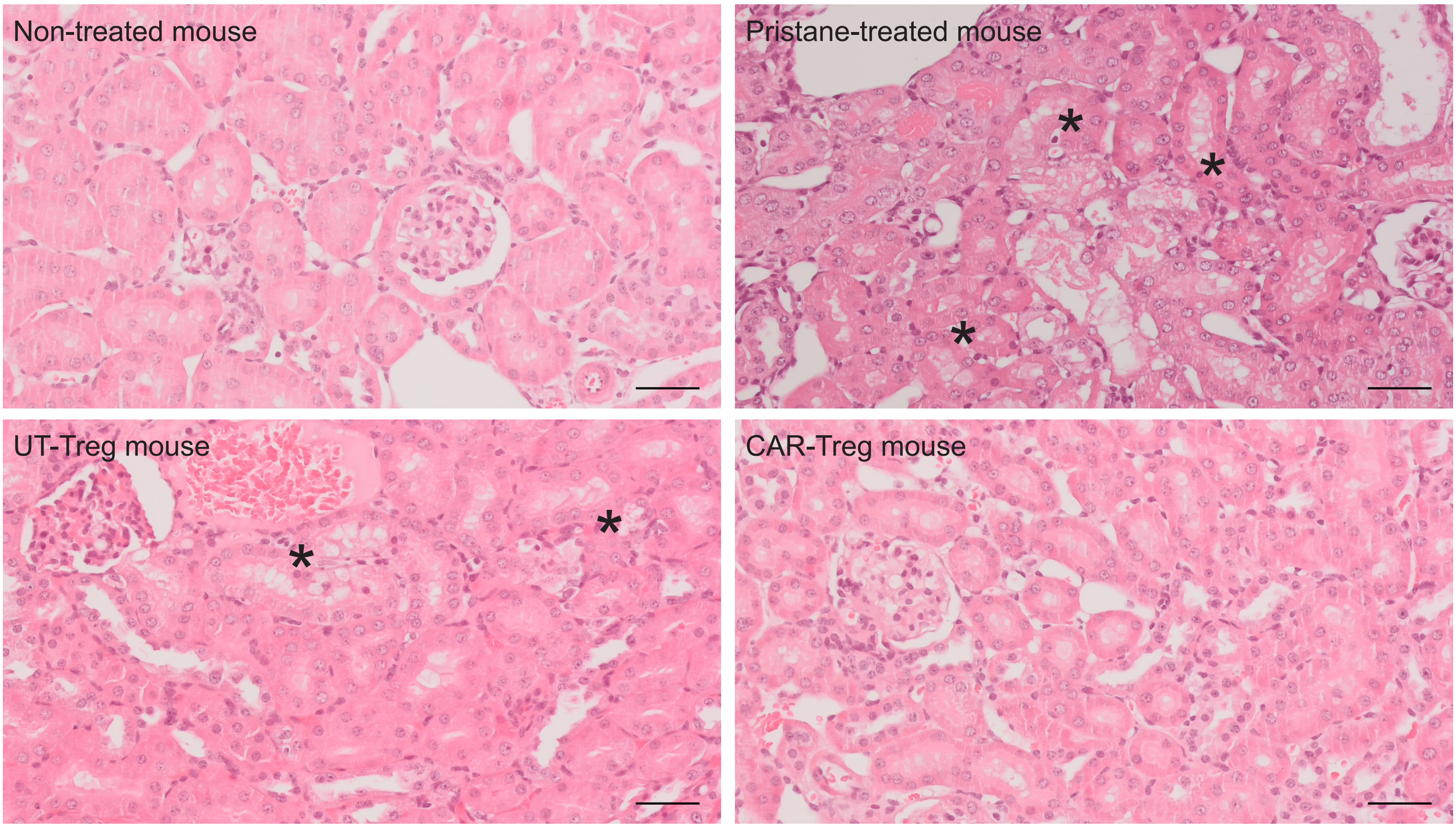
Non-treated mouse
Pristane-treated mouse
UT-Treg mouse
CAR-Treg mouse

D

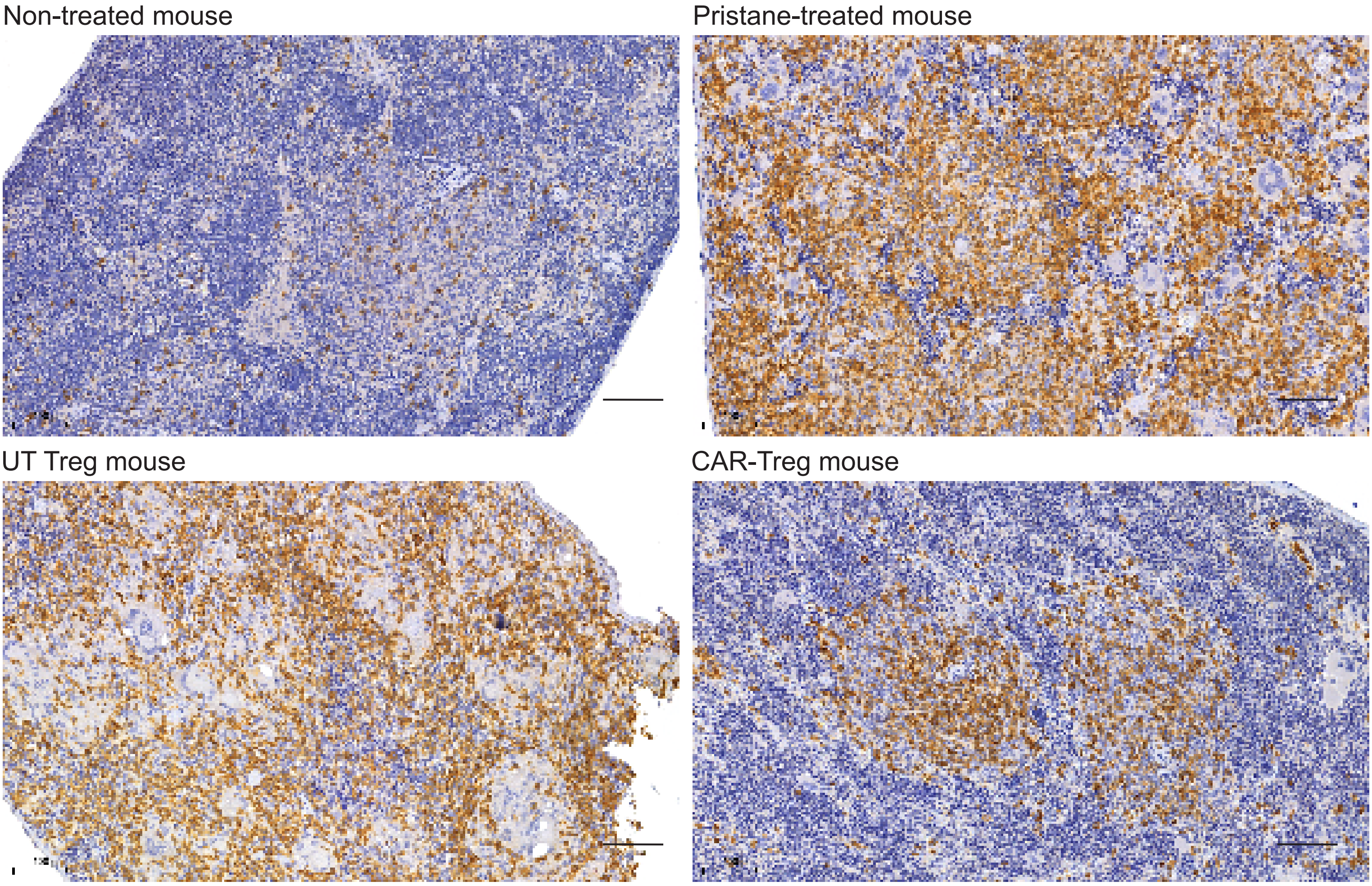
Non-treated mouse
Pristane-treated mouse
UT Treg mouse
CAR-Treg mouse

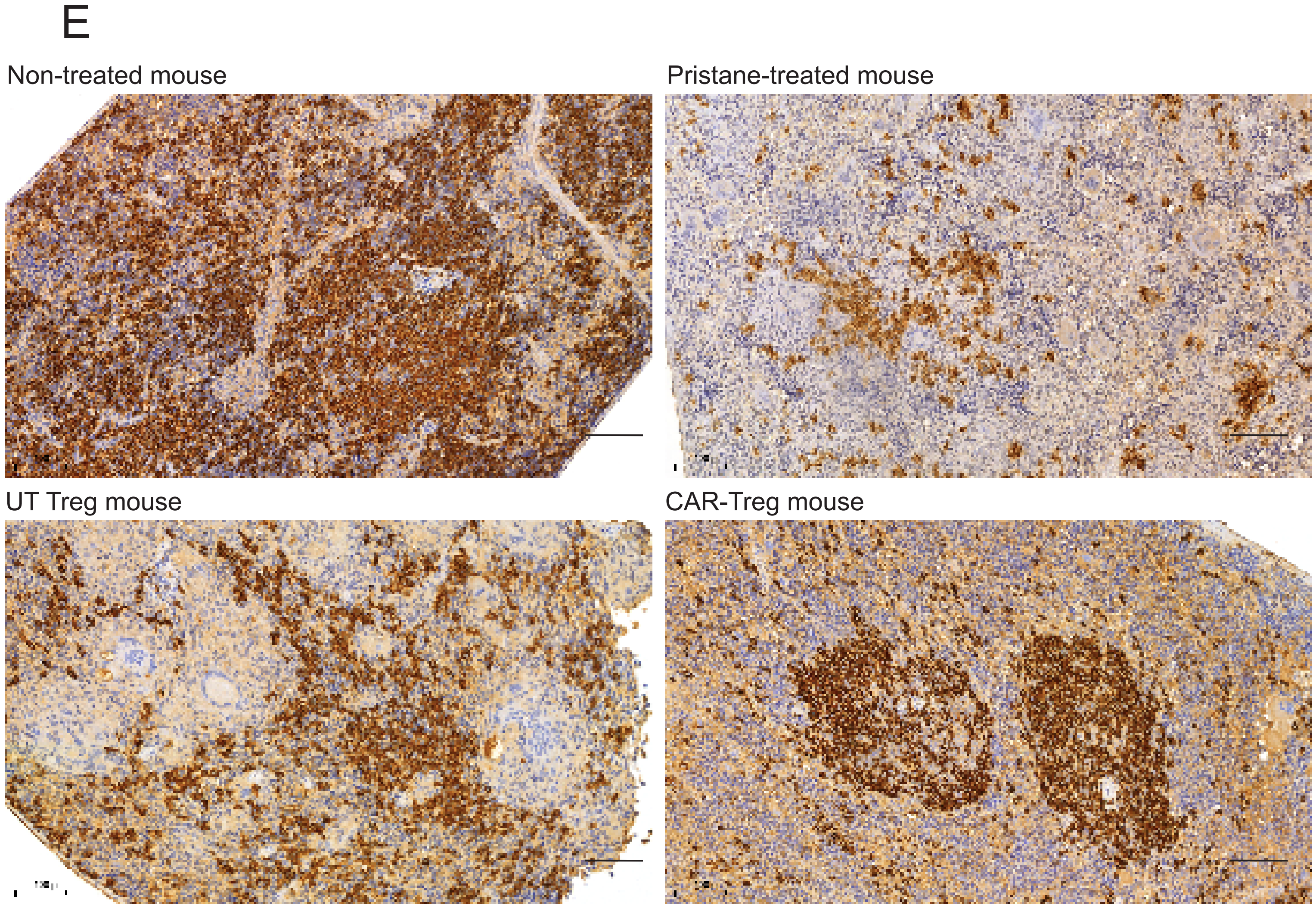

Supplementary Figure 6 – Human Treg phenotype and lymphoid organ composition in a humanized mouse model after treatment

A. Evaluation of the principal Treg-associated markers expressed by Tregs in the spleen, in the bone marrow and in kidneys. Their expression is reported as Mean Fluorescent Index (MFI). LAP, GARP = Glycoprotein A Repetitions Predominant, CTLA-4 = Cytotoxic T Lymphocyte Antigen 4, ICOS = Inducible Costimulator, GITR = Glucocorticoid-induced TNFR-related, TIGIT = T cell Immunoreceptor with Ig and ITIM domains. As control, humanized mice not treated with pristane (NT) are included. N=31 (14 CAR-Tregs, 8 UT-Tregs, 4 PBS, 5 NT). One-way ANOVA test with Tukey correction for multiple comparisons. * = p-value <0.05.

B. Percentage of total human CD45+ cells and human T and B cell sub-populations in kidneys at sacrifice. The results are expressed as mean ± standard deviation. As control, humanized mice not treated with pristane (NT) are included. N=38 (16 CAR-Tregs, 11 UT-Tregs, 5 PBS, 6 NT). One-way ANOVA test with Tukey correction for multiple comparisons. ** = p-value <0.01.

C. Representative pictures of the kidneys in 4 different mice, one per group of treatment. Asterisk indicates areas of tubular degeneration and vacuolization. Reference bar 100 μm.

D. Immunohistochemistry staining for human CD3 on the spleen, one per group of treatment. Reference bar 100 μm.

E. Immunohistochemistry staining for human CD20 on the spleen, one per group of treatment. Reference bar 100 μm.

Supplementary Figure 7

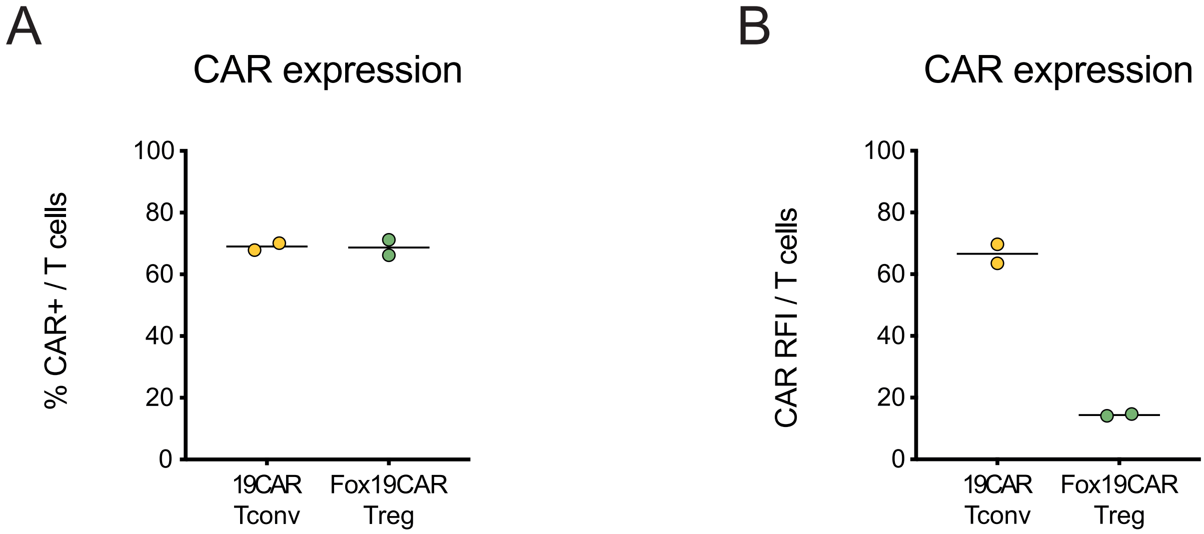

Supplementary Figure 7 – CAR expression in the cellular products employed in the SGM-3 based humanized mouse model

A-B. CAR expression in both Fox19CAR-Tregs and 19CAR-Tconvs, employed for the in vivo, assessed with the rCD19. N = 2 for each group.
