# Supplementary material for: Regulatory T cells expressing CD19-targeted chimeric antigen receptor restore homeostasis in Systemic Lupus Erythematosus
Source: Nat Commun. 2024 Mar 27;15:2542. doi: 10.1038/s41467-024-46448-9 (PMC10973480; doi:10.1038/s41467-024-46448-9)
Supplement: Supplementary file 3 — Reporting Summary [file 41467_2024_46448_MOESM3_ESM.pdf]

nature portfolio

Corresponding author(s): Prof Chiara Bonini, Dr. Matteo Doglio

Last updated by author(s): Feb 13, 2024

Reporting Summary

Nature Portfolio wishes to improve the reproducibility of the work that we publish. This form provides structure for consistency and transparency in reporting. For further information on Nature Portfolio policies, see our Editorial Policies and the Editorial Policy Checklist.

Statistics

For all statistical analyses, confirm that the following items are present in the figure legend, table legend, main text, or Methods section.

n/a	Confirmed	
☐	☒	The exact sample size (*n*) for each experimental group/condition, given as a discrete number and unit of measurement
☐	☒	A statement on whether measurements were taken from distinct samples or whether the same sample was measured repeatedly
☐	☒	The statistical test(s) used AND whether they are one- or two-sided<br>*Only common tests should be described solely by name; describe more complex techniques in the Methods section.*
☐	☒	A description of all covariates tested
☐	☒	A description of any assumptions or corrections, such as tests of normality and adjustment for multiple comparisons
☐	☒	A full description of the statistical parameters including central tendency (e.g. means) or other basic estimates (e.g. regression coefficient) AND variation (e.g. standard deviation) or associated estimates of uncertainty (e.g. confidence intervals)
☐	☒	For null hypothesis testing, the test statistic (e.g. *F*, *t*, *r*) with confidence intervals, effect sizes, degrees of freedom and *P* value noted<br>*Give P values as exact values whenever suitable.*
☒	☐	For Bayesian analysis, information on the choice of priors and Markov chain Monte Carlo settings
☒	☐	For hierarchical and complex designs, identification of the appropriate level for tests and full reporting of outcomes
☒	☐	Estimates of effect sizes (e.g. Cohen's *d*, Pearson's *r*), indicating how they were calculated

Our web collection on statistics for biologists contains articles on many of the points above.

Software and code

Policy information about availability of computer code

Data collection	BD diva software for flow cytometry analysis, G-power software for statistical power evaluation for in vivo experiments, RandoMice was employed to randomize the mice in the different experimental groups
Data analysis	Prism 10 for graphs and statistics, Flowjo 10 for flow cytometry, Cytochain for multiparametric flow cytometry

For manuscripts utilizing custom algorithms or software that are central to the research but not yet described in published literature, software must be made available to editors and reviewers. We strongly encourage code deposition in a community repository (e.g. GitHub). See the Nature Portfolio guidelines for submitting code & software for further information.

Data

Policy information about availability of data

All manuscripts must include a data availability statement. This statement should provide the following information, where applicable:

- Accession codes, unique identifiers, or web links for publicly available datasets
- A description of any restrictions on data availability
- For clinical datasets or third party data, please ensure that the statement adheres to our policy

The data that support the findings of this study are not openly available due to reasons of sensitivity and are available from the corresponding author upon reasonable request. Data are located in controlled access data storage at IRCCS San Raffaele Scientific Institute.

Research involving human participants, their data, or biological material

Policy information about studies with human participants or human data. See also policy information about sex, gender (identity/presentation), and sexual orientation and race, ethnicity and racism.

Reporting on sex and gender	Healthy Donors' samples have been collected anonymously and no informations were available regarding their sex and age, for this reason we haven't provided any data regarding sex and gender
Reporting on race, ethnicity, or other socially relevant groupings	Healthy Donors' samples have been collected anonymously and no informations were available regarding their sex and age, for this reason we haven't provided any data regarding race, ethnicity or social relevant groupings
Population characteristics	Healthy Donors' samples have been collected anonymously and no informations were available
Recruitment	Healthy Donors were recruited after the signature of an informed consent. Donors were collected blidly and no informations were available about their characteristics
Ethics oversight	This study complies all the required ethical requirements. The use of healthy donors' Peripheral Blood Mononuclear Cells was approved by the San Raffaele Ethical Committee. The in vivo studies involving mice were approved by the Italian Ministry of Health.

Note that full information on the approval of the study protocol must also be provided in the manuscript.

Field-specific reporting

Please select the one below that is the best fit for your research. If you are not sure, read the appropriate sections before making your selection.

☒ Life sciences ☐ Behavioural & social sciences ☐ Ecological, evolutionary & environmental sciences

For a reference copy of the document with all sections, see nature.com/documents/nr-reporting-summary-flat.pdf

Life sciences study design

All studies must disclose on these points even when the disclosure is negative.

Sample size	Sample size for in vivo studies was calculated with the G-power software to obtain a significant difference in circulating B cells, considering a power of 0.8 and type I error of 0.05.
Data exclusions	No data were excluded
Replication	For the in vitro experiments, minimum of 3 different donors were employed to replicate the results. The exact number of replicates is reported in each experiment.<br>For the in vivo experiments, different donor sources for both human hematopoietic stem cells and engineered cells were employed to enhance the reproducibility of the resuts. The exact number of the replicates is reported for each in vivo experiment in the manuscript and all were successful.
Randomization	Animals were randomly assigned to the various experimental group according to both sex and humanization level. RandoMice software was employed for the mouse randomization.<br>For in vitro experiments, the variables for each Healthy Donor were unavailable so we didn't perform any randomization
Blinding	The evaluation of the pathological specimens was performed in double-blind by a pathologist specifically trained in mouse pathology.<br>For the experiments, the variables for each Healthy Donor were unavailable so we didn't employ any blinding procedure

Reporting for specific materials, systems and methods

We require information from authors about some types of materials, experimental systems and methods used in many studies. Here, indicate whether each material, system or method listed is relevant to your study. If you are not sure if a list item applies to your research, read the appropriate section before selecting a response.

Materials & experimental systems

n/a	Involved in the study	
☐	☒	Antibodies
☐	☒	Eukaryotic cell lines
☒	☐	Palaeontology and archaeology
☐	☒	Animals and other organisms
☒	☐	Clinical data
☒	☐	Dual use research of concern
☒	☐	Plants

Methods

n/a	Involved in the study	
☒	☐	ChIP-seq
☐	☒	Flow cytometry
☒	☐	MRI-based neuroimaging

Antibodies

Antibodies used

For Treg phenotype, cells were labeled with titrated fluorescent monoclonal antibodies specific for CD3 (eFluor506, Invitrogen, OKT3, 69-0037-42), CD4 (PE-Vio615, Miltenyi, REA623, 130-113-226), FoxP3 (PE-Cy5, eBioscience, PCH101, 15-4776-42), CD45RA (BUV496, BD Bioscience, 5H9, 741182), CD62L (BUV737, BD Bioscience, SK11, 749210), TIGIT (BV786, BD Bioscience, 741182, 747838), LAP (Per-CP-eFluor710, eBioscience, FNLAP, 46-9829-42), Helios (PE, Biolegend, 22F6, 137216), CD25 (PE-Vio770, Miltenyi, REA945, 130-116-205), CD27 (APC, Biolegend, M-T271, 356410), CD137 (Alexa Fluor 700, Biolegend, 4B4-1, 309816), CD127 (APC-Vio770, Miltenyi, REA614, 130-113-416), GARP (BV421, BD Bioscience, 7B11, 563956), CTLA-4 (BV605, Biolegend, BNI3, 369610), ICOS (BV650, BD Bioscience, DX29, 563832), GITR (BV711, Biolegend, 108-17, 371212). For 19CAR engineered cells, transduction efficiency was assessed with GFP or NGFR spacer (CD271, BD Bioscience, clone C40-1457), conjugated in BB515 (564580), PE (557196) or PE-Cy7 (562122). Fox19CAR engineered cells were identified using biotinylated recombinant human CD19 reagent (Miltenyi, 130-129-550). Biotin was detected using VioBright515-conjugated anti-biotin secondary antibody (Miltenyi, Bio3-18E7, 130-113-298). For intra-nuclear staining, lymphocytes were stained with surface antibodies, washed, fixed and permeabilized with FoxP3 staining buffer set (Miltenyi, 130-093-142), according to manufacturer's instructions.
For in vivo experiments, whole blood was lysed with ACK (Ammonium-Chloride-Potassium) buffer for 10 minutes at room temperature to remove red blood cells. The reaction was then stopped with PBS supplemented with 5% FBS. Subsequently, samples were stained with recombinant CD19 reagent. After washing with PBS supplemented with 5% FBS, fluorochrome-conjugated monoclonal antibodies specific for mouse CD45 (PerCP, Biolegend, 30-F11, 103130), human CD45 (PE-Cy7, Invitrogen, HI30, 25-0459-42), CD3 (eFluor506, Invitrogen, OKT3, 69-0037-42), CD14 (APC-Cy7, Biolegend, 63D3, 367108), CD19 (APC, Biolegend, 4G7, 392504), CD56 (PE, Biolegend, 5.1H11, 362508) were added to samples. Human T cells were counted on peripheral blood using Flow Count fluorescent beads (Beckman Coulter), according to manufacturer's instruction.
For Treg analysis in harvested organs, single-cell suspensions were labeled with fluorochrome-conjugated monoclonal antibodies specific for CD3 (eFluor506, Invitrogen, OKT3, 69-0037-42), CD4 (PE-Vio615, Miltenyi, REA623, 130-113-226), FoxP3 (PE-Cy5, eBioscience, PCH101, 15-4776-42), CD45RA (BUV496, BD Bioscience, 5H9, 741182), CD62L (BUV737, BD Bioscience, SK11, 749210), TIGIT (BV786, BD Bioscience, 741182, 747838), LAP (Per-CP-eFluor710, eBioscience, FNLAP, 46-9829-42), Helios (PE, Biolegend, 22F6, 137216), CD25 (PE-Vio770, Miltenyi, REA945, 130-116-205), CD27 (APC, Biolegend, M-T271, 356410), CD137 (Alexa Fluor 700, Biolegend, 4B4-1, 309816), CD127 (APC-Vio770, Miltenyi, REA614, 130-113-416), GARP (BV421, BD Bioscience, 7B11, 563956), CTLA-4 (BV605, Biolegend, BNI3, 369610), ICOS (BV650, BD Bioscience, DX29, 563832), GITR (BV711, Biolegend, 108-17, 371212). Recombinant CD19 reagent (Miltenyi, 130-129-550) was used to detect CAR+ T cell, as previously described. Prior to the staining, mouse FC blocking reagent was employed according to the manufacturer's instructions (Miltenyi) to avoid specific binding and to reduce noise.
For immune cells infiltrating the harvested organs at sacrifice, single-cell suspensions were labeled with fluorochrome-conjugated monoclonal antibodies specific for human CD45 (PE-eFluor610, eBioscience, 2D1, 61-9459-42) CD3 (eFluor506, Invitrogen, OKT3, 69-0037-42), CD4 (BV785, Biolegend, RM4-5, 100552), CD19 (FITC, Biolegend, 4G7, 392508), CD20 (PE-Cy7, Biolegend, 2H7, 302312), CD138 (PE-Cy5, BeckmanCoulter, B-A38, A54191), CD27 (APC-Cy7, Biolegend, M-T271, 356424), CD14 (PerCP, Biolegend, M5E2, 301824), HLA-DR (BUV805, BD Bioscience, G46-6, 748338), PD-1 (BV650, Biolegend, EH12.2H7, 329950), CD163 (BV605, BD Bioscience, GHI/61, 745091), CD56 (PE, Biolegend, 5.1H11, 362508), CD206 (APC, Biolegend, 15-2, 321110).
For the in vivo studies, human leukocytes were defined as human CD45+ cells. T cells were defined as CD3+ cells. B cell subsets were defined as hCD45+CD19+ lymphocytes. B cell sub-populations were defined as: pre-B cells CD19+CD20-CD27- cells, naïve B cells CD19+CD20+CD27- cells, memory B cells CD19+CD20+CD27+ cells, plasmablasts CD19+CD20-CD27+ cells, plasma cells CD138+ cells. For each experiment, dead cells were excluded by DAPI positive staining. The amount of each antibody has been titrated for each lot as suggested on the data sheet, before the use.

Validation

Each primary and secondary antibody was specifically titrated on either primary cells or cell lines as suggested by the vendor on the datasheet. The optimal antibody amount was assessed evalutaing the Stain Index and the Separation Index

Eukaryotic cell lines

Policy information about cell lines and Sex and Gender in Research

Cell line source(s)

Commercial ALL-CM cell line was employed for some in vitro functional studies. This establishment of this cell line is reported in Bondanza et al. Blood 2011
Human peripheral blood mononuclear cells were employed, derived from healthy volunteers.
CD34+ human hematopoietic stem cells were derived from anonymous healthy subjects, whose informations were unavailable, and purchased from Lonza and certified by the vendor

Authentication

ALL-CM cell line was not furhter authenticated

Mycoplasma contamination	Mycoplasma contamination was checked routinely before in vitro and in vivo experiments by PCR and every time it resulted negative
Commonly misidentified lines (See ICLAC register)	No commonly misidentified cell lines were employed

Animals and other research organisms

Policy information about studies involving animals; ARRIVE guidelines recommended for reporting animal research, and Sex and Gender in Research

Laboratory animals	We employed 1-day-old NSG mice and 8-weeks-old adult SGM-3 mice.<br>Animals were kpet in germ-free conditions. Temperature was 20°C +/- 4°C. Humidity 55% +/- 10%. Dark/light cycles of 12 hours each. Ventilation: 8-10/cycles per hour and 75 cycles in ventilated cages
Wild animals	No wild animals were employed
Reporting on sex	Mouse sex was considered in the randomization process to homogenize treatment groups
Field-collected samples	No field-collected samples were employed
Ethics oversight	The study protocol was approved by both the San Raffaele Institutional Ethical Committee for animal studies and Italian Ministry of Health

Note that full information on the approval of the study protocol must also be provided in the manuscript.

Plants

Seed stocks	N/A
Novel plant genotypes	N/A
Authentication	N/A

Flow Cytometry

Plots

Confirm that:

☒ The axis labels state the marker and fluorochrome used (e.g. CD4-FITC).

☒ The axis scales are clearly visible. Include numbers along axes only for bottom left plot of group (a 'group' is an analysis of identical markers).

☒ All plots are contour plots with outliers or pseudocolor plots.

☒ A numerical value for number of cells or percentage (with statistics) is provided.

Methodology

Sample preparation	For in vitro experiments, human Treg and Tconv cells were washed with flow cytometry washing buffer, stained for the extracellular antigens at room temperature for 15 minutes and then washed again with the washing buffer and acquired. For intra-nuclear staining, lymphocytes were stained with surface antibodies, washed, fixed and permeabilized with FoxP3 staining buffer set (Miltenyi), according to manufacturer's instructions.<br>For in vivo experiments, whole blood was lysed with ACK (Ammonium-Chloride-Potassium) buffer for 10 minutes at room temperature to remove red blood cells. The reaction was then stopped with PBS supplemented with 5% FBS. Subsequently, samples were stained for the extracellular antigens at room temperature for 15 minutes and then washed again with the washing buffer and acquired.<br>For the analysis of immune cells in harvested organs, single-cell suspensions were stained for the extracellular antigens at room temperature for 15 minutes and then washed again with the washing buffer and acquired.<br>For the Treg analysis in harvested organs, single-cell suspensions were washed with flow cytometry washing buffer, stained for the extracellular antigens at room temperature for 15 minutes and then washed again. Cells were subsequently fixed and permeabilized with FoxP3 staining buffer set (Miltenyi), according to manufacturer's instructions, stained for intracellular antigens and then acquired.

Instrument	BD Canto II and BD Symhony A5 were employed
Software	BD Diva software was employed for the data acquisition. Flowjo 10 was employed for the data analysis
Cell population abundance	CD4+CD25+ cells were isolated by magnetic cell separation according to the manufacturer's instructions (Miltenyi). Mean post-sorting purity was 94-96% and was assessed by flow cytometry
Gating strategy	For the analysis of the Treg phenotype both in vitro and in vivo, live CD3+ cells were isolated. On this population, CD4+CD25+ cells were selected. Subsequently CD127+ cells were excluded and on CD127- cells FoxP3 and CAR percentages were assessed.<br>For in vivo experiments, live human CD45+ cells were initially isolated. On this population, the percentages of the various subpopulations were assessed.

☒ Tick this box to confirm that a figure exemplifying the gating strategy is provided in the Supplementary Information.
